# Surgical Management of Peripheral Ossifying Fibroma: A Report of Two Cases

**DOI:** 10.7759/cureus.69190

**Published:** 2024-09-11

**Authors:** Ruchi Pandey, Pooja Palwankar

**Affiliations:** 1 Periodontology, Manav Rachna Dental College, School of Dental Sciences, Manav Rachna International Institute of Research and Studies, Faridabad, IND

**Keywords:** electrocautery, electrocoagulation, fibroma, non neoplastic lesion, peripheral ossifying fibroma, surgical method

## Abstract

A nonneoplastic lesion of uncertain origin most likely occurring due to connective tissue cells of the periodontal ligament (PDL) is referred to as peripheral ossifying fibroma (POF). It has an exorbitant recurrence rate; therefore, proper diagnosis based on clinical, radiological, and histopathological investigations is a must along with an appropriate treatment plan, and follow-up of the patient is very important to avoid further recurrence. In the present case series, there was a recurrence of the lesion in the first case, a surgical method using a scalpel to excise the tissue with ample curettage till the surface of the bone was felt, followed by electrocoagulation. In the second case, surgical excision was done using a scalpel, and electrocautery was used to arrest bleeding. There was no recurrence reported till one year of follow-up.

## Introduction

Reactive gingival lesions are attributed mostly to systemic causes, iatrogenic factors, drug-related, and most commonly linked to dental plaque. It can progress in size which may take from weeks to months. The prevalence rate of reactive lesions of gingiva based on studies of Zhang et al. in 2439 patients and Lazare et al. in 41 cases reported that peripheral fibroma is 56%-61%, pyogenic granuloma is 19%-27%, peripheral ossifying fibroma (POF) is 10%-18%, and peripheral giant cell granuloma is 1.5%-7% [1-2]. Lesions show gender predilection, occurring more frequently in females (except peripheral giant cell granuloma), mostly in the anterior region and having a slight predilection for the maxilla [3]. POF was described first by Menzel as ossifying fibroma in 1872, but it was termed by Montgomery in 1927 [4]. POF can be further subclassified primarily into two: central and peripheral. The first one grows in the medullary compartments of the bone and emerges from the endosteum of the bone or the periodontal ligament (PDL) next to the root apex, whereas the second one is observed to arise from the soft tissues covering the alveolar process [5]. A single, slowly growing nodule mass that might be sessile or stalked is known as a POF, typically seen between neighboring teeth's gingival papilla [6]. The etiology of POF has a lot of uncertainty; it is suggested that PDL cells are the primary cause for its occurrence; this is attributed to the fact that most POF occur mostly from the interdental papillary region and oxytalan fibers are present within the mineralized matrix of lesion, along with fibrillar, cellular response in PDL [5-6].

Few cases have shown that there is migration of teeth due to interdental bone loss. On orthopantomogram (OPG), most of cases have shown no radiographic evidence of bone loss other than few cases, where superficial bone loss is evident. Deep excision of the lesion is necessary to prevent any reappearance since the lesions exhibit reappearance, which is mostly related to inadequate. This case series comprises of two cases with POF.

## Case presentation

Case 1

**Figure 1:**
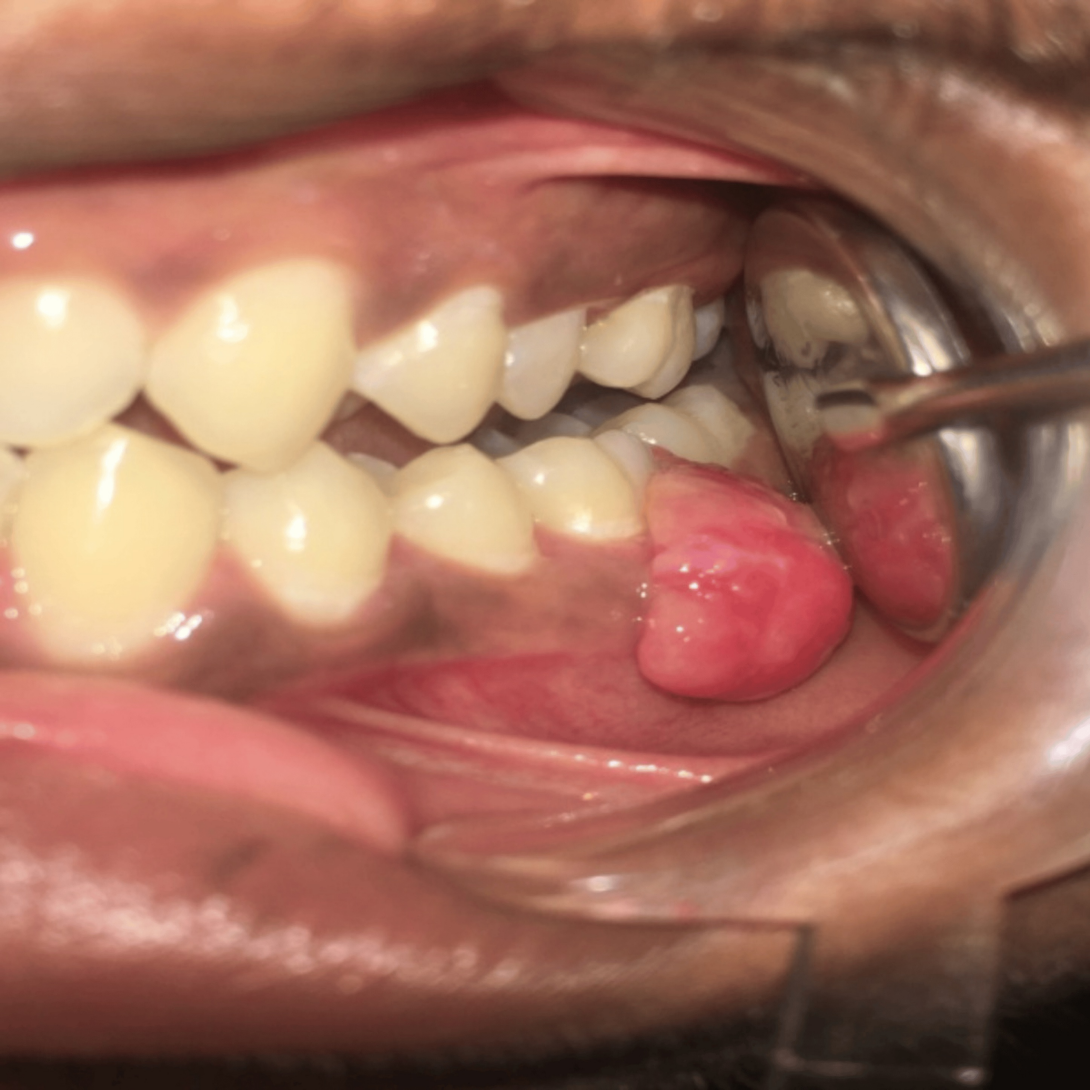
Preoperative view of case 1 Lesion from #35-37

A male patient of 22 years came to the outpatient department (OPD), complaining about swelling associated with the interdental area in the lower left back tooth region for two months. He was systemically healthy and did not have any harmful oral habits. The patient's dental history dates back to two years when he noticed a growth over his gums in the left lower back tooth region. This growth was removed elsewhere and has recurred in the last two months. The lesion was initially small and nurdle-sized and had attained a larger size. No history of pain was recorded. The lesion was almost of the same color as that of the adjacent gingiva, i.e., reddish-pink and was dome-shaped, nontender, non-ulcerated, pedunculated, and firm in consistency, almost obliterating the buccal vestibule. The swelling was 2 × 1 cm extending from mesial of 35 to distal of 37 (Figure 1).

Secondary changes like ulceration and fungation were not present, and the pathology was compacted and not painful on palpation. The growth was not fluctuant and could not be reduced in size and neither was compressible but bled mildly on probing.

**Figure 2:**
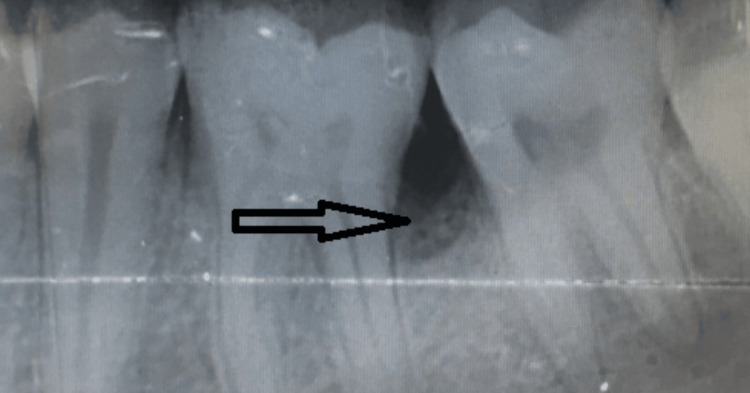
Preoperative radiograph of case 1 The arrow indicates saucer-shaped defect in relation to #36-37

The provisional clinical diagnosis was given as irritational fibroma with respect to the 35 to 37 region. Radiologically, the intraoral periapical radiograph (IOPA) of the left mandibular posterior area showed the appearance of a bone defect (saucer-shaped) in relation to 36 and 37. A faint, asymmetrical opacity was noticed in between the 36 and 37 region suggestive of evident loss of bone in the area (Figure 2).

**Figure 3:**
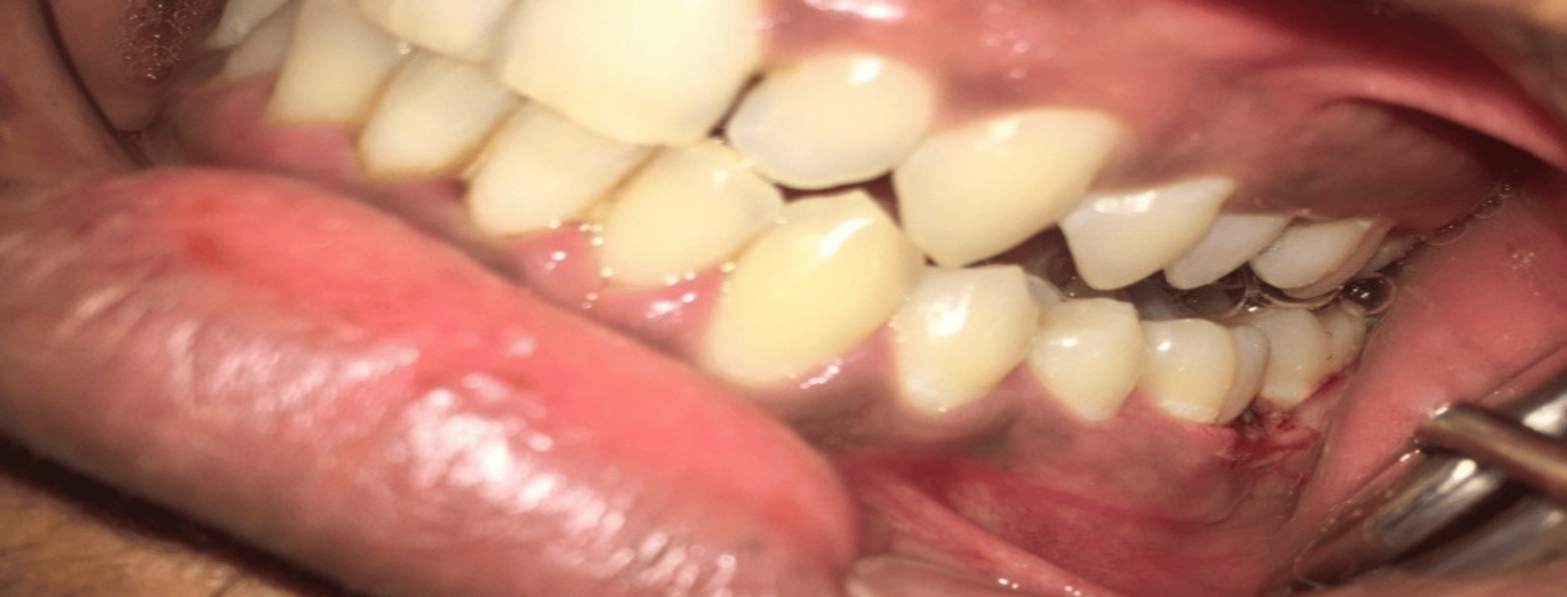
Postoperative view of case 1

The pathology was extirpated along with some normal tissue using the scalpel method under local anesthesia (LA) containing 2% lignocaine hydrochloride with adrenaline 1:80000 (Xicaine), before the intraoral use LA sensitivity test was done. The underlying surface was deeply curetted up to the deepest possible tissue, i.e., the bone (Figure 3).

**Figure 4:**
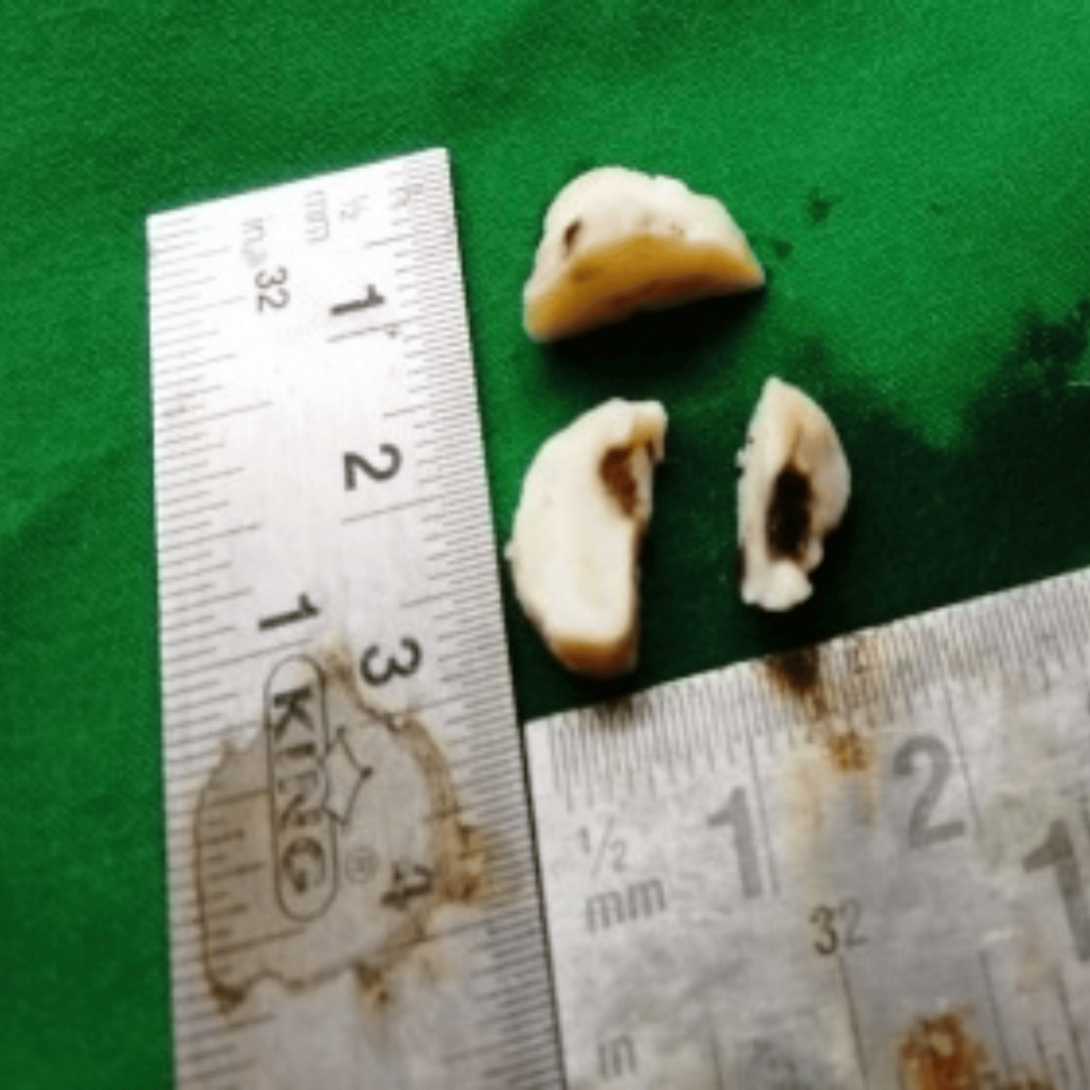
Dimensions of biopsy sample of case 1

Bleeding was controlled using electrocautery, and the tissue was put in 10% neutral buffer formalin for biopsy. The tissue on excision was ovoid, sized nearly about 2 × 1 cm, whitish brown in color, and gritty in consistency (Figure 4).

Excised tissue in solution of 10% formalin was sent for histological analysis. Post-excision, routine periodontal therapy was performed, and regular follow-up for the patient was done.

**Figure 5:**
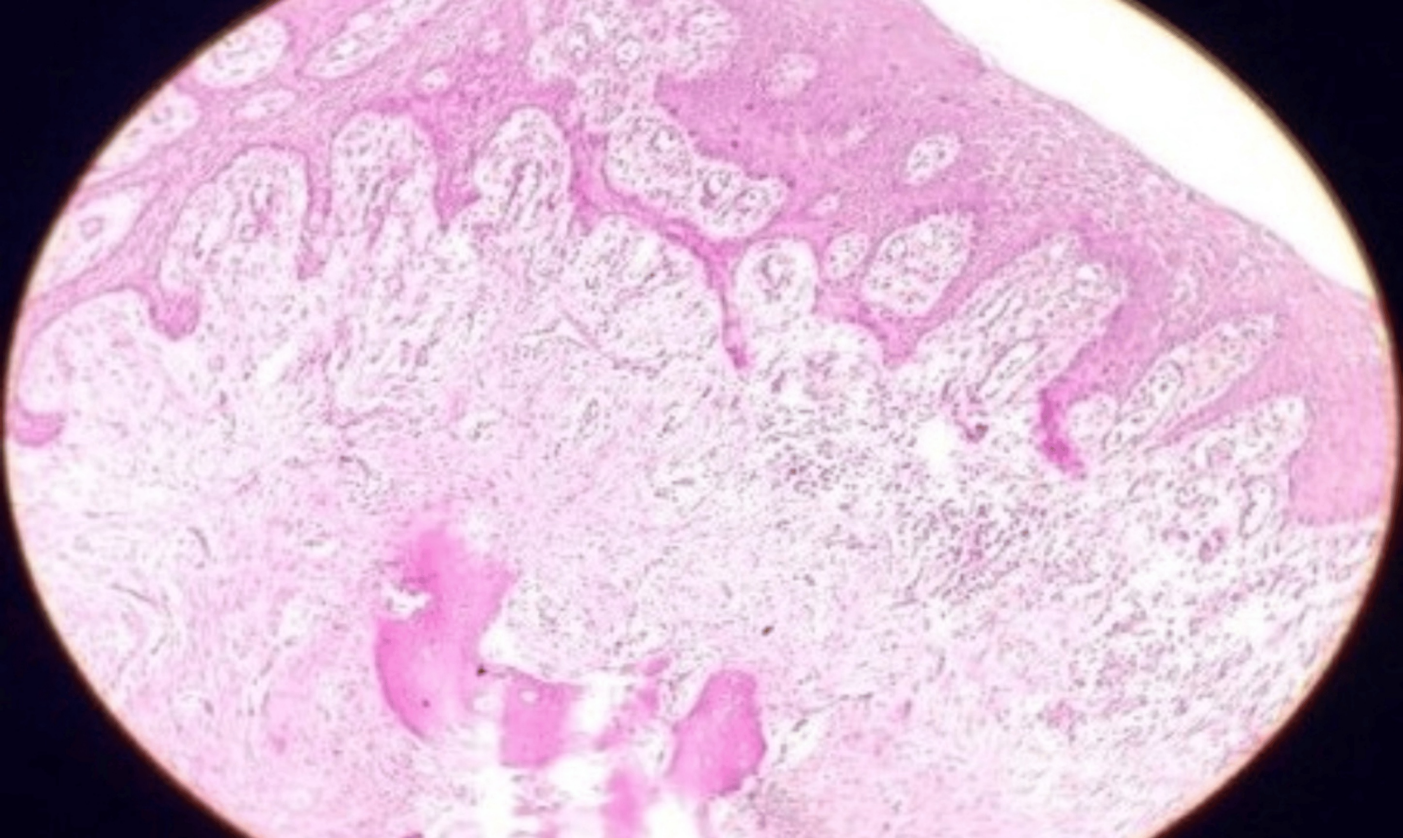
Histopathological slide of case 1

The tissue sample was stained with hematoxylin and eosin (H/E) staining and showed cellular fibrous connective tissue stroma made up of plump fibroblasts, mixed inflammatory cell infiltrate, sparse endothelial proliferation, several irregular trabeculae of bone, osteoid, and few dystrophic calcifications. The stratified squamous epithelium that covers is hyperplastic and para keratinized and has anastomosing rete pegs that are thin, lengthy, and ulcerated in certain areas (Figure 5).

Therefore, the histopathological diagnosis was POF. The patient's follow-up for one year showed no reappearance.

Case 2

**Figure 6:**
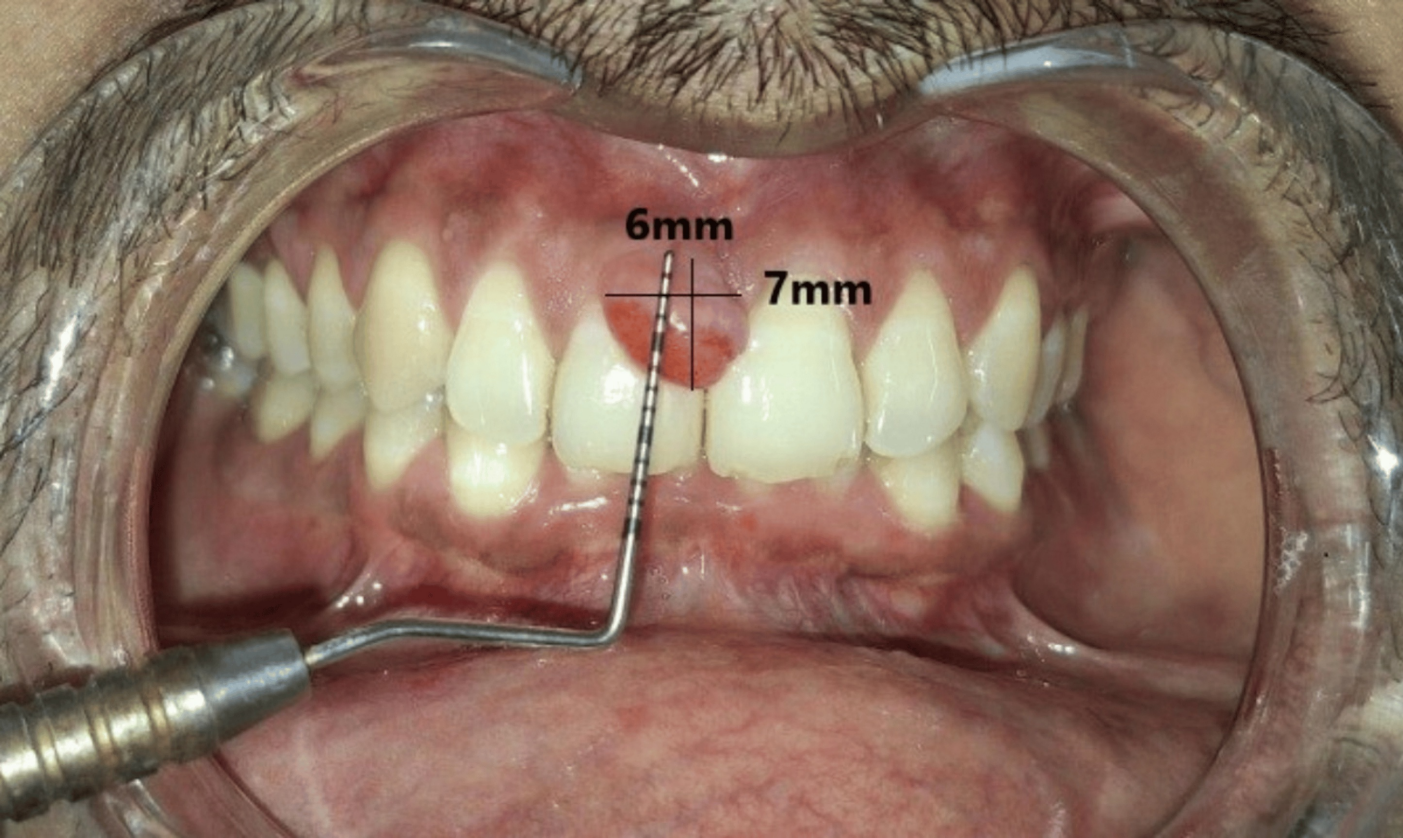
Preoperative view of case 2

A male patient of 33 years, systemically healthy, delineated his complaint as swelling in the gums in the front tooth region for two weeks. The patient was well before two weeks when he had an injury from a toothbrush and the interdental papillary gingiva kept on increasing in size with time. It was pedunculated, non-ulcerated, painless, and compacted in nature. The pathology was 6 × 7 × 3 mm in size, extending from distal 11 to mesial 21 (Figure 6).

**Figure 7:**
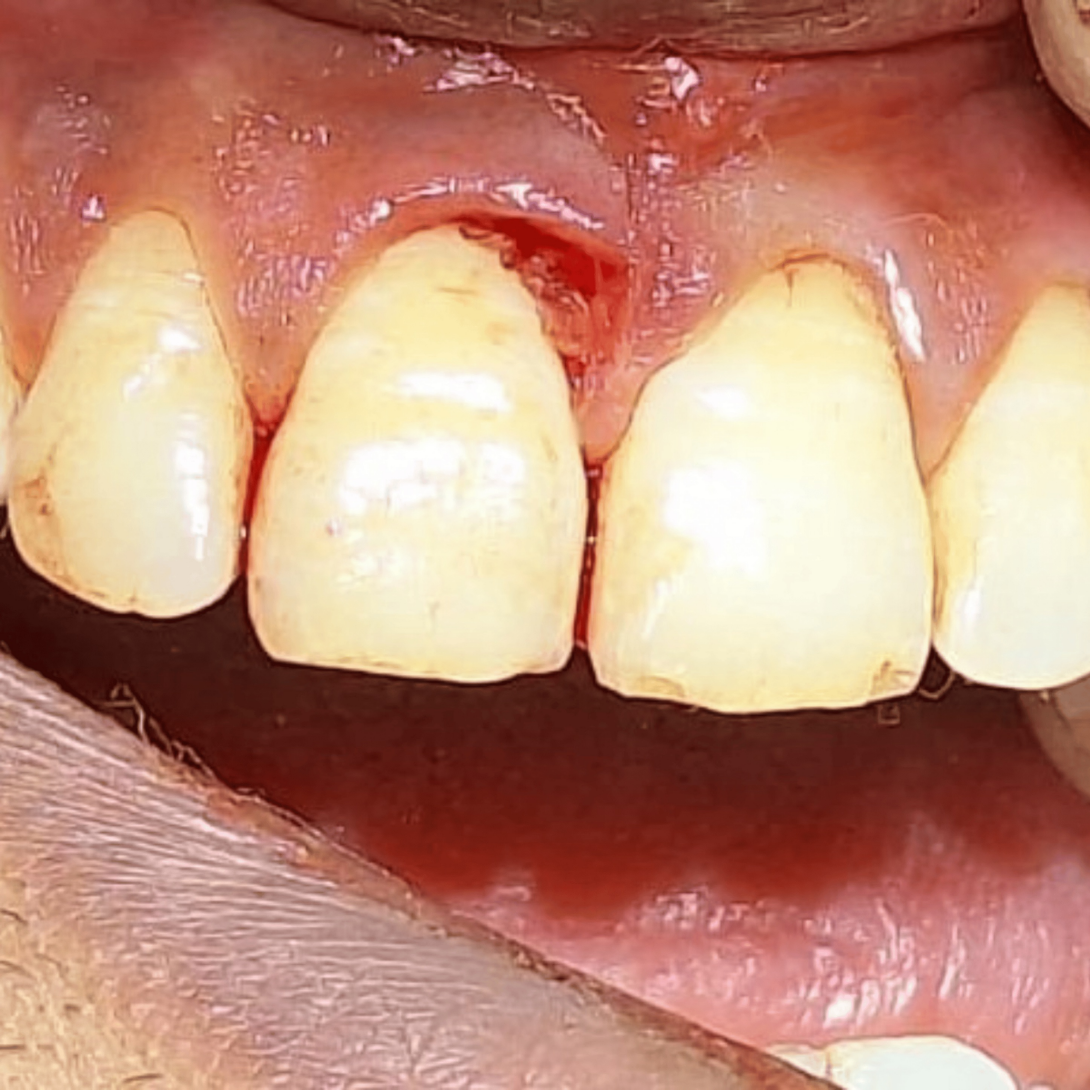
Postoperative view of case 2

The lesion was extirpated using a scalpel and electrocautery and sent for histopathological analysis (Figure 7).

**Figure 8:**
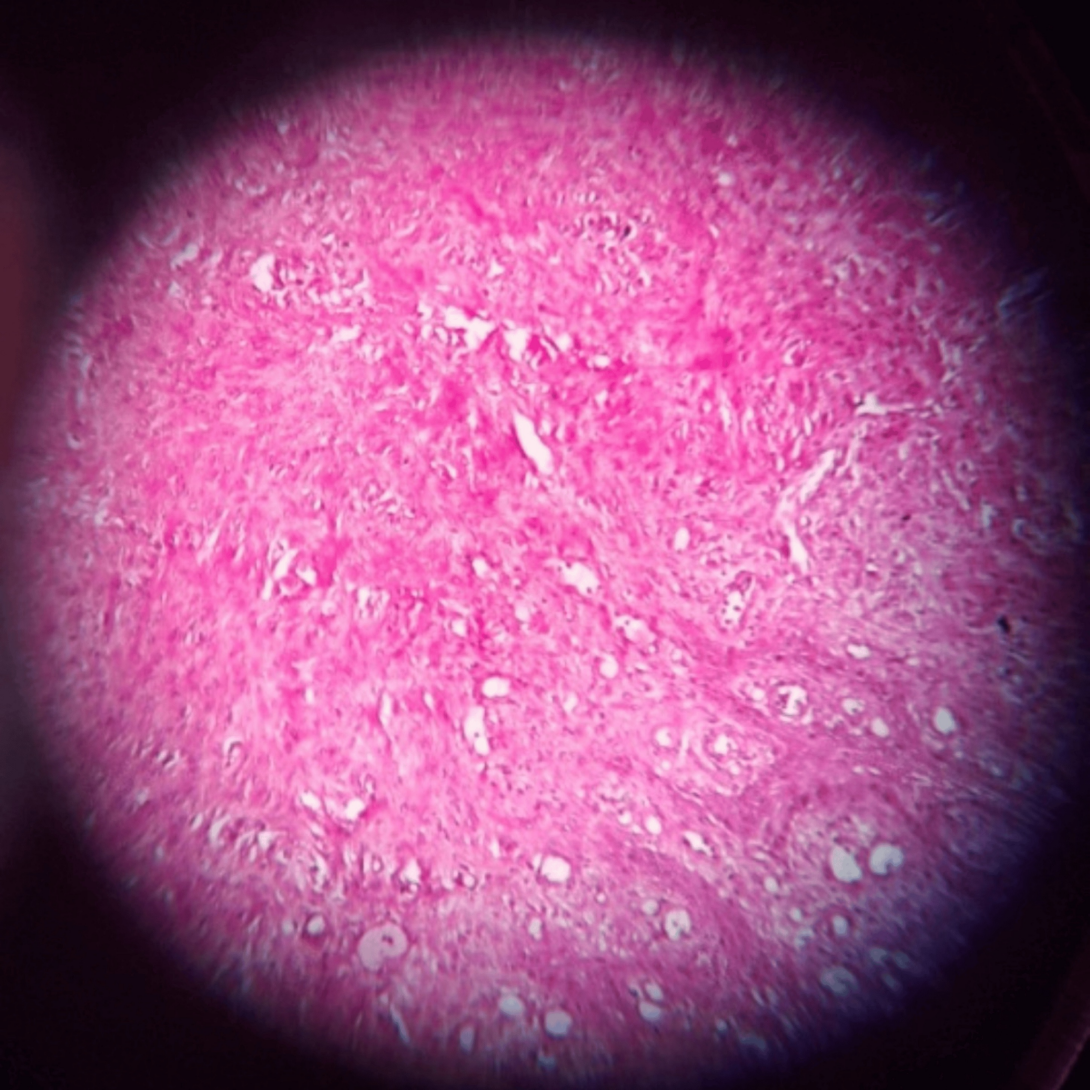
Histopathological slide for case 2

The provisional diagnosis was given as epulis, and the biopsy report revealed POF (Figure 8). The patient showed no signs of recurrence even after two years of stringent follow-up.

## Discussion

It is important to differentiate POF from other lesions like pyogenic granuloma, traumatic fibroma, and peripheral giant cell granuloma. Another reactive lesion of the gingiva is pyogenic granuloma, which is extremely vascular and gives the lesion a reddish-purple color. It is frequently raised and ulcerated and bleeds at the slightest provocation. Long-standing lesions appear pink due to the presence of more collagen bundles. Vascular spaces are lined with endothelium infiltrated with cells like lymphocytes, plasma cells, and neutrophils. Extensive proliferation of fibroblastic cells is present which are diffuse and often accompanied by dense chronic inflammatory infiltrate. A thin, often ulcerated stratified squamous epithelial membrane covers the lesion. Despite its name, pyogenic granuloma is devoid of pus or pyogenic material. The other differential diagnosis for POF is traumatic fibroma; other names for it are irritational fibroma, focal fibrous hyperplasia, or fibrous nodule. It is a commonly occurring reactive lesion, originating from stimulated connective tissue of the gingiva. The etiology of the lesion is trauma or chronic irritation. Lesion resembles a raised mass of soft tissue which may be sessile or pedunculated, having a smooth shiny surface almost similar to the surrounding gingiva. Traumatic fibroma as the name suggests is fibrous with a varying degree of vascularity. According to histopathology, the lesion is entirely coated with keratinized squamous cell epithelium and is not ulcerated. A traumatic fibroma consists of bundles of collagen fibers, which are often arranged in various patterns, i.e., circularly, radially, and irregularly. The majority of such lesions have a thick layer of fibrous connective tissue that is poorly supplied by blood, containing relatively few cells of chronic inflammation.

Peripheral giant cell granuloma originates in the periosteum or PDL cells; etiology remains the same as other reactive pathology, i.e., local irritation/chronic trauma. Lesions exhibit color from deep red to reddish blue, stalked or unstalked, occurring routinely in the lower jaw. Although the lesion can develop at any age, it has been shown that the fourth and sixth decades of life are the most common. A nonencapsulated tissue mass made up of reticular and fibrillar connective tissue with a profusion of stout ovoid or spindle-shaped multipotent cells is what the lesion looks like upon histological inspection. The keratinized squamous cell epithelium, which may exhibit a break in continuity due to ulcerations, covers the peripheral giant cell granuloma. The lesion's predominant characteristic is the abundance of dispersed, multinucleated giant cells.

POF is a reactionary lesion defined as “any sole growth on the gingiva” expected to develop from the cell rests of Malassez, seen mostly in females in the second decade of life [7]. It originates from the gingiva and accounts for 9.6% of gingival lesions [8]. Commonest at the interdental papillary region, occurring due to persistent abuse of periosteal and periodontal membrane leading to metaplasia and dystrophic calcifications resulting in fibrosis of granulation tissue [9], hormones may play a role in these changes therefore seen more in female subjects [7]. POF is highly cellular, usually exhibiting bone, with mixed inflammatory cell infiltrate, sparse endothelial cell proliferation, and irregularly arranged bony trabeculae with few dystrophic calcifications. Its recurrence rate reported by Cundiff is 16% and a study by Eversole and Robin reported recurrence as 20% [3,6]. The recurrence rate varies from 8.9% to 20%, and the causative factor is a failure of extirpation of the pathological lesion completely, regular irritation due to injury, or incomplete removal of locally present irritating factors. On average, the first recurrence of the lesion is seen between 12 and 14 months [10].

In case 1, the etiology of the lesion can be attributed to local irritants, such as biofilm, subgingival calculus, and traumatic occlusion. The patient in the case report gave a history of recurrent lesions in the interdental area of 36 and 37. On intraoral examination, a nodular/gritty mass, on palpation it was firm, stalked, 2 cm in size, with a reddish pink color. On radiological examination, intraoral mass presented with a radiolucent area (in IOPA). After the clinical, and radiological examination and correlating with histological features, it was concluded that the lesion was POF. In case 2, the causative factor is attributed to irritation caused by toothbrush bristles.

## Conclusions

POF is a reactionary lesion of the gingiva. It is often misinterpreted and can be wrongly diagnosed with other reactionary lesions of the gingiva. A thorough clinical and histopathological examination must be done to arrive at a proper diagnosis and its management. Its clinicopathological presentation is similar yet diverse. There is a significant overlap between the different types of focal reactive gingiva overgrowths. Clinical-pathological features can change in contrast to how POF is often presented.

To accurately evaluate and manage patients, a differential diagnosis must be made to identify any reactive lesions. Lesions tend to recur if inappropriately excised, thus to prevent recurrence, total removal of pathology and aggressive curetting of the adjacent tissues becomes mandatory.
